# *Acer palmatum thumb.* Ethanol Extract Alleviates Interleukin-6-Induced Barrier Dysfunction and Dextran Sodium Sulfate-Induced Colitis by Improving Intestinal Barrier Function and Reducing Inflammation

**DOI:** 10.1155/2018/5718396

**Published:** 2018-10-08

**Authors:** Kwang-Youn Kim, Tae-Woo Oh, Hyun Ju Do, Ju-Hye Yang, In Jun Yang, Yong Hyun Jeon, Young-Hoon Go, Soon-Cheol Ahn, Jin-Yeul Ma, Kwang-Il Park

**Affiliations:** ^1^Korean Medicine (KM) Application Center, Korea Institute of Oriental Medicine (KIOM), Daegu 41062, Republic of Korea; ^2^Department of Physiology, College of Korean Medicine, Dongguk University, Gyeongju 38066, Republic of Korea; ^3^Laboratory Animal Center, Daegu Gyeongbuk Medical Innovation Foundation, Daegu 41061, Republic of Korea; ^4^Department of Microbiology and Immunology, Pusan National University School of Medicine, Yangsan 50612, Republic of Korea

## Abstract

Ulcerative colitis is one inflammatory bowel disease (IBD) and is caused by diverse factors, including the extent and duration of intestinal inflammation. We investigated the effect of *Acer palmatum thumb.* ethanol extract (KIOM-2015E) on the expression of tight junction proteins and the levels of inflammation in the cell model induced with interleukin-6- (IL-6-) and mouse model of dextran sodium sulfate (DSS) induced with acute colitis. KIOM-2015E (100 mg/kg) was orally administered once per day to BALB/C mice with colitis induced by administration of 5% DSS in drinking water. KIOM-2015E did not affect viability in Caco-2 cells. Also, KIOM-2015E repaired the IL-6-induced intestinal barrier dysfunction in Caco-2 cells. Furthermore, KIOM-2015E recovered the loss of body weight and the abnormally short colon lengths in the DSS-induced model of acute colitis. Moreover, KIOM-2015E significantly inhibited the decrease of zonula occluden-1 and occludin in colonic tissue relative to the DSS-treated control group. KIOM-2015E also significantly inhibited the expression of IL-6 and tumor necrosis factor-*α* in the level of serum relative to the control group. Collectively, these data suggest that KIOM-2015E protects colitis principally by improving intestinal barrier function and promoting anti-inflammatory responses. In turn, these effects inhibit macrophage infiltration into the colon and thus may be a candidate treatment for IBD.

## 1. Introduction

Inflammatory bowel disease (IBD) has two main forms including ulcerative colitis (UC) and Crohn's disease (CD) in human [1]. Symptoms of UC and CD include abdominal pain, diarrhea, and/or fever [2]. Also, IBD is caused by environmental interactions and genetic and lifestyle factors [3]. Many evidence demonstrated that one of the most typical character of IBD is the abnormal immune response within the intestinal wall by luminal bacterial antigens following epithelial barrier disruption [2]. The epithelial junction complex comprised tight junction (TJ) proteins of epithelial cells, such as zonula occluden-1 (ZO-1), occludin, and claudins, which are related with intracellular complex and cytoskeleton [4, 5]. These TJ proteins play an important role in forming and maintaining the paracellular barrier, but destruction of these functions was associated with initiation and acceleration of the inflammation of IBD [6, 7]. Recently, therapies of IBD have used anti-inflammatory drugs, immune modulators, and surgery. However, these therapies focused on controlling the immune cell response, including the cytokine pathway, but have many serious side effects including allergies and carcinoma [8]. Therefore, several researchers have been focusing on reliable data regarding the efficacy and safety to improved intestinal barrier function using natural or traditional herbs [9].

*Acer palmatum Thumb* is widely distributed as ornamentals in diverse regions including Asia, North America, North Africa, and Europe, and it has been used in traditional medication in East Asia [10, 11]. The roots, leaves, husks, stems, and fruits of maple are used as herbal medicines for treatment with detoxification, relieving rheumatism and bone pain, reducing sore throat, improving eyesight, and reducing digestion [11]. Especially, stems and roots of maple has been used for the treatment of arthritis, bruises, pain, fractures, and liver diseases and the leaves are used for the treatment of inflammation of the eye [10, 11]. However, the intracellular mechanism of Acer palmatum Thumb underlying the efficacy of colitis, inflammation, and damage to tight junctions remains unclear. Therefore, we investigated whether ethanol extract of *Acer palmatum Thumb* (KIOM-2015E) can alleviate acute colitis and evaluated the protective effects using the DSS-induced mouse colitis model.

## 2. Material and Methods

### 2.1. Reagents

The extract named KIOM-2015E, which is the extract of *Acer palmatum Thumb*, has been described in our previous research [12]. Dextran sodium sulfate (DSS), 5-aminosalicylic acid (5-ASA), and hematoxylin and eosin solutions were purchased from MP Biomedicals (Santa Ana, CA, USA) and Sigma Aldrich (St Louis, MO, USA). The ELISA kits of myeloperoxidase (MPO) activity, mouse tumor necrosis factor-alpha (TNF-*α*), and interleukin-6 (IL-6) were purchased from Thermo Fisher Scientific (Waltham, MA, USA) and eBioscience (San Diego, CA, USA). RIPA lysis buffer and phosphatase and protease inhibitor cocktails were obtained from Millipore (Darmstadt, Germany) and Roche (Basel, Switzerland), respectively. A BCA protein quantification kit, fluorescence-tagged antibody and anti-zonula occludens-1 (ZO-1), anti-occludin antibody, and anti-F4/80 antibody were purchased from Thermo Fisher Scientific and Santa Cruz Biotechnology (Dallas, TX, USA).

### 2.2. Cell Culture

Caco-2 cells (American Type Culture Collection, Manassas, VA, USA) were cultured in Eagle's Minimum Essential Media (HyClone, Logan, UT, USA) supplemented with 10% fetal bovine serum (HyClone), 100 U/ml penicillin, and 100 *μ*g/ml streptomycin (HyClone) at 37°C in an atmosphere of 5% CO_2_.

### 2.3. Cell Viability

Cell viability was evaluated by Cell Counting Kit-8 (CCK-8) (Dojindo Molecular Technologies Inc., Rockville, MD, USA). Briefly, Caco-2 cells were plated at 1 × 10^3^ cells/well in a 96-well plate. Cells were pretreated with KIOM-2015E for 1 h and then treated with IL-6 for an additional 24 h. CCK-8 assay was then performed in accordance with the manufacturer's instructions. Absorbance was determined at 450 nm on a VersaMax microplate reader (Molecular Devices, Sunnyvale, CA, USA). Cell viability was calculated relative to untreated controls (i.e., viability (% control) = 100 × absorbance of treated sample/absorbance of control).

### 2.4. Measurement of Transepithelial Electrical Resistance (TER)

Caco-2 cells were seeded at 1 × 10^5^ cells/insert in 0.33 cm^2^ polyethylene terephthalate membrane inserts with 0.4 *μ*m pores (Millipore, Bedford, MA). The medium was changed every 2 days until complete differentiation. Cells were pretreated with KIOM-2015E for 1 h and then treated with IL-6. The electrical resistance was measured for three independent measurements using by Millicell ERS-2 Voltohmmeter (Millipore). TER was obtained at 18 and 24 h and presented as Ohm·cm^2^.

### 2.5. Epithelial Paracellular Permeability

Caco-2 cells were seeded at 1 × 10^5^ cells/insert in 0.33 cm^2^ polyethylene terephthalate membrane inserts with 0.4 *μ*m pores (Milipore). The medium was changed every 2 days until complete differentiation. Cells were pretreated with KIOM-2015E for 1 h and then treated with IL-6 for an additional 24 h. The paracellular permeability was evaluated using a nonabsorbable, FITC-conjugated dextran probe (FD-4). Following pretreatment, the apical and basolateral sections were washed with PBS and then added to 1 mg/ml FD-4 in the apical side and PBS in the basolateral side. Following 1 h of incubation at 37°C, 100 *μ*l of media from the basolateral side was plated to a 96-well plate The excitation and emission wavelengths were determined at 490 and 520 nm on a VersaMax microplate reader (Molecular Devices).

### 2.6. Animals

All animal experimental procedures were approved by the Korea Institute of Oriental Medicine Institutional Animal Care and Use Committee (KIOM-D-17-023) and were conducted in accordance with the guidelines of the National Institutes of Health (NIH publication number 83-23, revised in 1985). Male C57/BL6 mice (6 weeks) were purchased from Samtako Inc. (Osan, Korea), divided into 5 groups: vehicle-treated control (*n* = 10), KIOM-2015E 100 mg/kg (*n* = 10), 5% DSS (*n* = 10), 5% DSS + KIOM-2015E 100 mg/kg (*n* = 10), and 5% DSS + 5-ASA 100 mg/kg (*n* = 10). During the experimental schedule, the mouse body weight was measured daily before oral administration of KIOM-2015E. The colonic lengths were measured after the animals were sacrificed by photographs.

### 2.7. Large Intestine Endoscopy and Histological Analysis

On 8 days of the experiment, we performed endoscopy using a mini-endoscope (OLYMPUS, Tokyo, Japan; 670 mm length and 2.8 mm diameter) with a visible light source, to investigate the colons of mice anesthetized with isoflurane, and we obtained high-resolution images. After the endoscopy procedure, whole blood was obtained from the mice abdominal vein. And the animals were then sacrificed for intestinal tissue collection. The isolated colons were fixed with 4% paraformaldehyde solution, embedded in paraffin block, and sectioned using a microtome. Histological sections were stained with hematoxylin and eosin (H&E), alcian blue, or PAS solution or incubated with antibodies to detect the macrophage marker, F4/80, and the tight junction protein, ZO-1.

### 2.8. Cell Culture and Establishment of a Stable Cell Line

The murine macrophage Raw264.7 cell line was obtained from ATCC (Manassas, VA, USA). Cells were cultured at 37°C in a humidified atmosphere with 5% CO_2_ in Dulbecco's modified Eagle's medium (HyClone) containing 10% fetal bovine serum (HyClone), 100 unit/ml penicillin, and 100 *μ*g/ml streptomycin (HyClone). Cells were lentivirally transfected to express with both human sodium iodide symporter (hNIS) as a single nuclear reporter gene and green fluorescent protein (GFP) genes. The GFP-positive cells were sorted by flow cytometry (FACSaria; BD Biosciences, San Jose, CA, USA). And then cells were further retrovirally transduced to express both enhanced firefly luciferase (effluc) and Thy1.1 gene to stabilize expressed hNIS. Thy1.1-positive cells were sorted using CD90.1 microbeads (Miltenyi Biotec, Auburn, CA, USA) and sorted by flow cytometry to determine the stable expression of the GFP and hNIS genes using a PercP-conjugated Thy1.1 antibody (BD Biosciences). These cells were named the Raw264.7/NF cell line in the present study [13].

### 2.9. In Vivo Image for Macrophage Infiltration Using Bioluminescence Imaging (BLI)

To monitor for infiltration of macrophage in DSS-induced colitis mice, we performed in vivo imaging systems (IVIS). On the 7th day of experiment, the mice were injected in the tail intravenously with 100 *μ*l of Raw264.7/NF cells (3 × 10^6^ cells/mouse). On the 8th day of experiments, the mice were injected intraperitoneally with 100 *μ*l of D-luciferin (3 mg/mouse). For the acquisition of BLI, the mice were placed individually in the specimen chamber of the IVIS system and the light intensity at 10 min after luciferin administration was measured for 1 min. BLI images were analyzed using Living Image (version 2.12, Xenogen, Alameda, CA, USA) and IGOR Image Analysis FX software (WaveMetrics, Portland, OR, USA). BLI signals were evaluated by drawing uniformly sized ROIs throughout the whole experiment and indicated in units of photons per square centimeter per second per steradian [13].

### 2.10. Enzyme-Linked Immunosorbent Assay for Myeloperoxidase (MPO) Activity, IL-6, and TNF-*α*

Collected whole blood was incubated at 4°C overnight and then centrifuged (3000 ×g at 4°C) for 15 min. The separated serum was stored at −80°C until use. The levels of IL-6 and TNF-*α* in the serum were determined using ELISA kits according to the manufacturer's protocol. In addition, MPO, a marker for neutrophil infiltration, was determined using MPO activity assay kit according to the manufacturer's protocol.

### 2.11. Western Blot Analysis

Caco-2 cells and mouse intestinal protein were extracted using RIPA lysis buffer with phosphatase and protease inhibitor cocktails. Equal proteins of quantification with a BCA kit were electrophoretically separated by 8% and 12% SDS-PAGE gels and then transferred to PVDF membranes. After blocking, the membranes were incubated with a 1 : 1000 dilution of ZO-1 or occludin antibody at 4°C overnight. The following day, the membranes were incubated with secondary antibodies and detected using the ChemiDoc Touch Imaging System (Bio-Rad, Hercules, CA, USA). The band density was then normalized to the *β*-actin reference.

### 2.12. Statistical Analysis

All statistical analyses were performed with SPSS version 18, and graphs were drawn with GraphPad Prism version 5. Experimental values are given as the means ± standard error of the mean. The significant difference was determined by one-way ANOVA test. *p* values less than 0.05 were regarded as statistically significant.

## 3. Results

### 3.1. Effects of IL-6 and KIOM-2015E on Cell Viability

**Figure 1:**
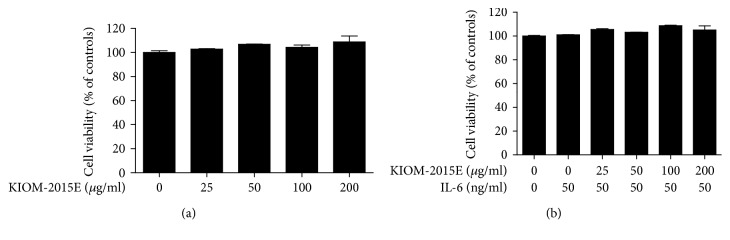
Effects of IL-6 and KIOM-2015E on cell viability of Caco-2 cells. (a) Cell viability of KIOM-2015E. (b) Cell viability of KIOM-2015E with interleukin-6 (IL-6). Cells were pretreated with various concentrations (25–200 *μ*g/ml) of KIOM-2015E for 1 h and then treated with 50 ng/ml IL-6 for 24 h. Cell viability was determined using a Cell Counting Kit-8. Cell viability is represented as the percentage relative absorbance compared with the controls. The results represent the mean ± standard error of the mean values of three independent experiments.

We determined the effects of IL-6 and KIOM-2015E on cell viability in Caco-2 cells. Cell viability was measured following treatment with various concentrations of KIOM-2015E (25–200 *μ*g/ml) and incubation for 24 h. These results showed that KIOM-2015E with/without IL-6 did not affect the cell viability in the Caco-2 cells (Figures 1(a) and 1(b)).

### 3.2. Effects of KIOM-2015E on IL-6-Induced Epithelial Barrier Dysfunction in Caco-2 Cell Monolayers

**Figure 2:**
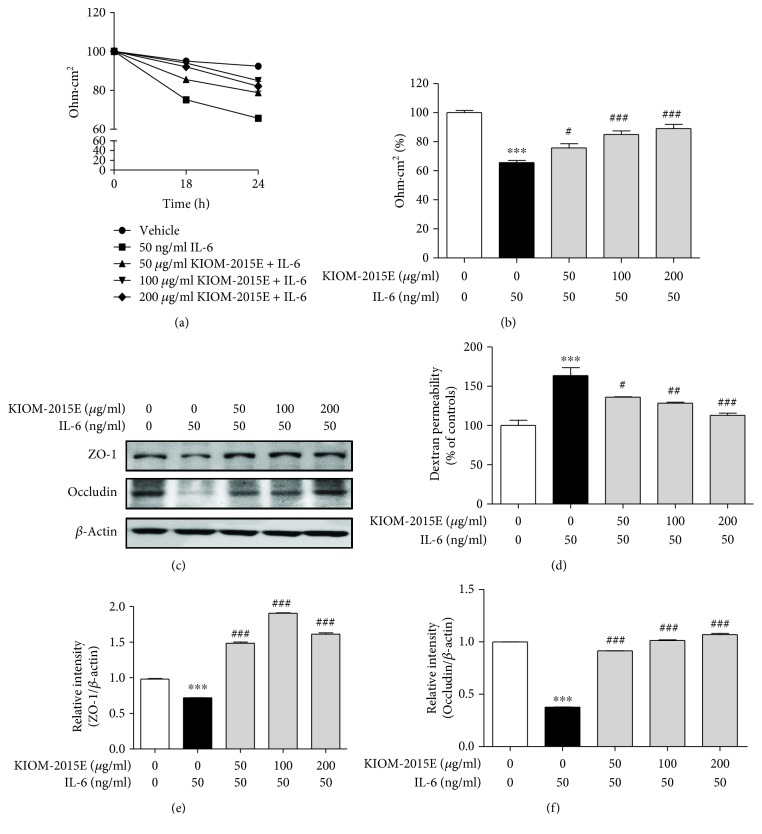
Effects of KIOM-2015E on Caco-2 monolayer intestinal barrier function. (a and b) Transepithelial electrical resistance (TER). (c) Epithelial paracellular permeability. Intestinal barrier function was analyzed via the TER value, and paracellular permeability was determined using a nonabsorbable, fluorescein isothiocyanate- (FITC-) conjugated dextran probe (FD-4). Caco-2 monolayer cells were pretreated with various concentrations (25–200 *μ*g/ml) of KIOM-2015E for 1 h and then treated with 50 ng/ml IL-6 exposure for 24 h. (d) Representative expression for ZO-1 and occludin proteins. Expression of ZO-1 and occludin in Caco-2 cells was determined by Western blotting. *β*-Actin was used as the protein-loading control. Densitometry of protein expression. (e) ZO-1. (f) Occludin. The results represent the mean ± standard error of the mean values of three independent experiments. ^∗∗∗^*p* < 0.001 compared with the control group; ^#^*p* < 0.05, ^##^*p* < 0.01, and ^###^*p* < 0.001 compared with the IL-6 treated group.

To determine the epithelial barrier dysfunction, we measured the flux of FITC dextran and TER. Previous reports showed that IL-6 could be decreased in TER (increased in permeability) in 50 ng/ml, with a maximal decrease in TER at 24 h [14]. Pretreatment with KIOM-2015E resulted in a significant increase in TER in a time- and dose-dependent manner compared with IL-6 treatment (Figures 2(a) and 2(b)). Also, pretreatment with KIOM-2015E decreases the FD-4 permeability in a dose-dependent manner compared with IL-6 treatment (Figure 2(c)). Furthermore, treatment with IL-6 decreased the tight junction-associated protein expression, namely, ZO-1and occludin; however, pretreatment with KIOM-2015E induced recovery (Figures 2(d)–2(f)). Our findings also indicate that KIOM-2015E may more obviously protect intestinal function via IL-6-mediated barrier dysregulation.

### 3.3. Effects of KIOM-2015E on DSS-Induced Loss of Body Weight, Colon Length, and MPO Activity

**Figure 3:**
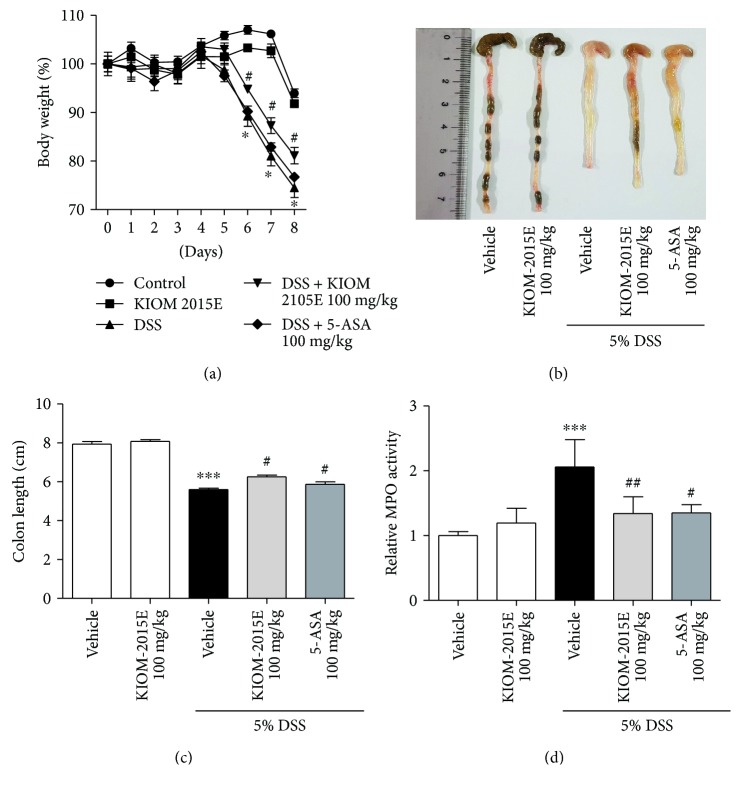
Effects of KIOM-2015E on body weight, colon length, and myeloperoxidase (MPO) activity in the dextran sodium sulfate- (DSS-) induced colitis model. (a) Body weight. (b and c) Colon length. (d) Myeloperoxidase (MPO) activity. Mice were orally administered KIOM-2015E or 5-aminosalicylic acid (5-ASA) (100 mg/kg) before DSS treatment. The body weights were monitored before KIOM-2015E or 5-ASA treatment during the experimental periods. The colonic lengths of mice were measured after being isolated from the sacrificed mice. The MPO activity was measured using MPO activity assay kit according to the manufacturer's protocol. The results represent the mean ± standard error of the mean values of each mouse in the same group. ^∗∗∗^*p* < 0.001 versus the control group; ^#^*p* < 0.05 and ^##^*p* < 0.01 versus the DSS-treated group.

We verified the therapeutic effect of orally administered KIOM-2015E on the severity of DSS-induced colitis. Compared with the vehicle control group, DSS-treated mice were significantly reduced body weight (Figure 3(a)). However, the administration of KIOM-2015E obviously improved the body weight loss as compared with the DSS-treated group (Figure 3(a)). Furthermore, consistent with body weight results, colon length shortened was markedly increased by administration of KIOM-2015E compared with that of the DSS-treated group (Figures 3(b) and 3(c)). In addition, DSS-treated mice had significantly higher MPO activity than those of the KIOM-2015E-treated group and the control group (Figure 3(d)).

### 3.4. Therapeutic Effects of KIOM-2015E in DSS-Induced Colitis Mice

**Figure 4:**
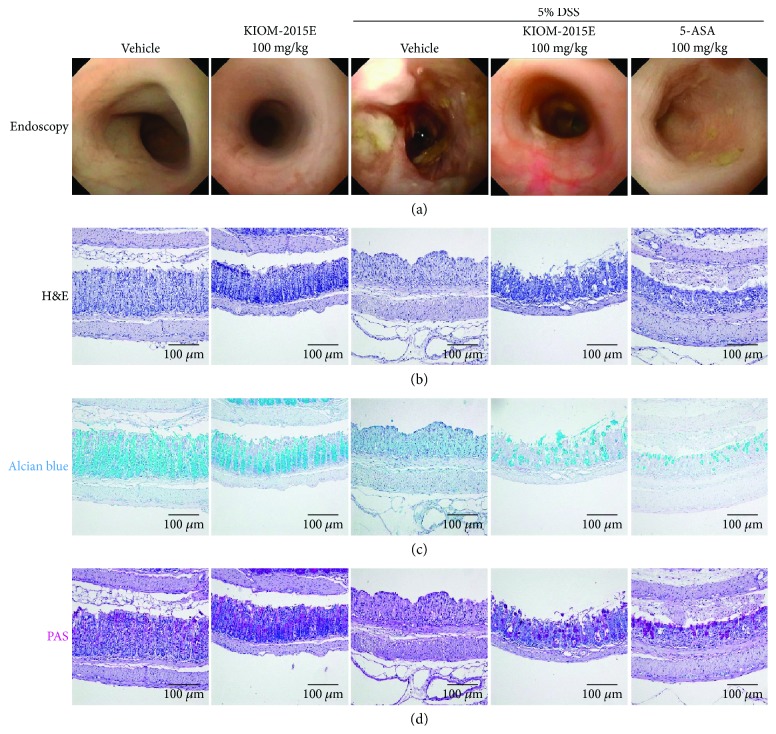
The protective effect of KIOM-2015E in the dextran sodium sulfate- (DSS-) induced colitis model. (a) Endoscopy image. (b) Hematoxylin and eosin staining (H&E) staining. (c) Alcian blue staining. (d) PAS staining. On the 8th day of experiments, we confirmed the DSS-induced mucosal damage using a mini-endoscope. After the endoscopy procedure, collected intestinal tissues were fixed with 4% paraformaldehyde solution, embedded in paraffin block, and sectioned using a microtome. Histological sections were stained with hematoxylin and eosin (H&E), alcian blue, and PAS solution to determined histological change. Represent images were shown. Magnification was 200x.

To determine the degree of colitis and the effects of KIOM-2015E, we evaluated the histological and morphological changes in the large intestine after the administration of DSS and KIOM-2015E by endoscopy, H&E, alcian blue, and PAS staining. The DSS-treated group has shown serious morphological and histological changes including ulcers of the colon membrane in colon tissue damage (Figure 4; endoscopy and H&E staining). However, this damage was repaired by administration of KIOM-2015E compared with the DSS-treated group at day 8 (Figure 4; endoscopy and H&E staining). Furthermore, the number of goblet cells was significantly reduced in the DSS-treated group (Figure 4; alcian blue and PAS staining). However, KIOM-2015E prevented the loss of goblet cells compared with the DSS group. These results suggest that KIOM-2015E could ameliorate against DSS-induced colitis (Figure 4; alcian blue and PAS staining).

### 3.5. Effects of KIOM-2015E on Intestinal Barrier Function in DSS-Induced Colitis Mice

**Figure 5:**
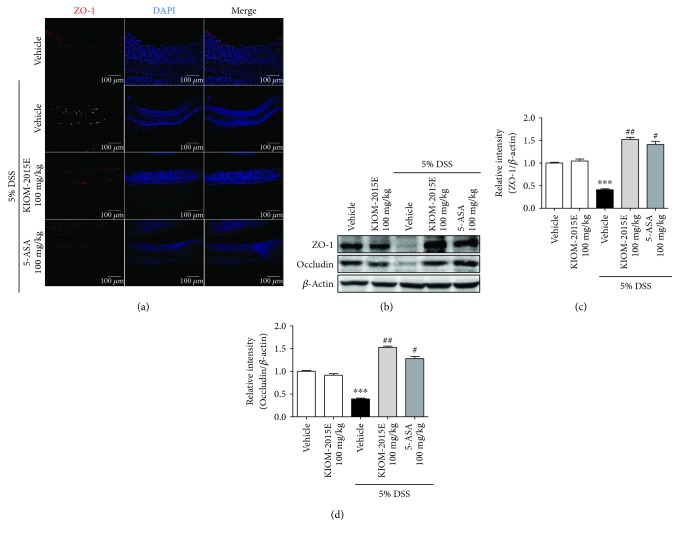
The protective effect of KIOM-2015E on tight junctions in the dextran sodium sulfate- (DSS-) induced colitis model. (a) Immunofluorescence staining for ZO-1 expression. Collected intestinal tissues were fixed with 4% paraformaldehyde solution, embedded in paraffin block, and sectioned using a microtome. Histological sections were stained with anti-ZO-1 (red) antibody and DAPI (blue) staining. (b) Representative expression for ZO-1 and occludin proteins. Expression of ZO-1 and occludin in colonic tissues was determined by Western blotting. *β*-Actin was used as the protein-loading control. (c and d) Densitometry of protein expression. (c) ZO-1. (d) Occludin. These Western blot data expressed the mean ± standard error of the mean values of each mouse in the same group. ^∗∗∗^*p* < 0.001 versus the control group; ^#^*p* < 0.05 and ^##^*p* < 0.01 versus the DSS-treated group.

Intestinal epithelial tight junctions have an important role in protecting against inflammation. Also, tight junction regulated the paracellular permeability by the forming of apical junction complex. Dysfunction of tight junction causes the disruption of the intestinal barrier and induced inflammation, following the pathogenesis of colitis [15]. Therefore, the present study evaluated the effects of KIOM-2015E on the protein expression of ZO-1 and occludin by immunofluorescence and Western blot analysis. ZO-1 expression was reduced after DSS treatment, and this downregulation was increased by treatment with KIOM-2015E (Figure 5(a)). Moreover, the decreased expression of ZO-1 and occludin in DSS-induced colitis was recovered by KIOM-2015E treatment (Figures 5(b)–5(d)). Together, these results indicated that KIOM-2015E prevented the downregulation of tight junction proteins in the DSS-induced acute colitis model and it might be relative with the preventive mechanism of colitis.

### 3.6. Effects of KIOM-2015E on Infiltration of Macrophages and Serum Levels of Proinflammatory Cytokines in DSS-Induced Colitis Mice

**Figure 6:**
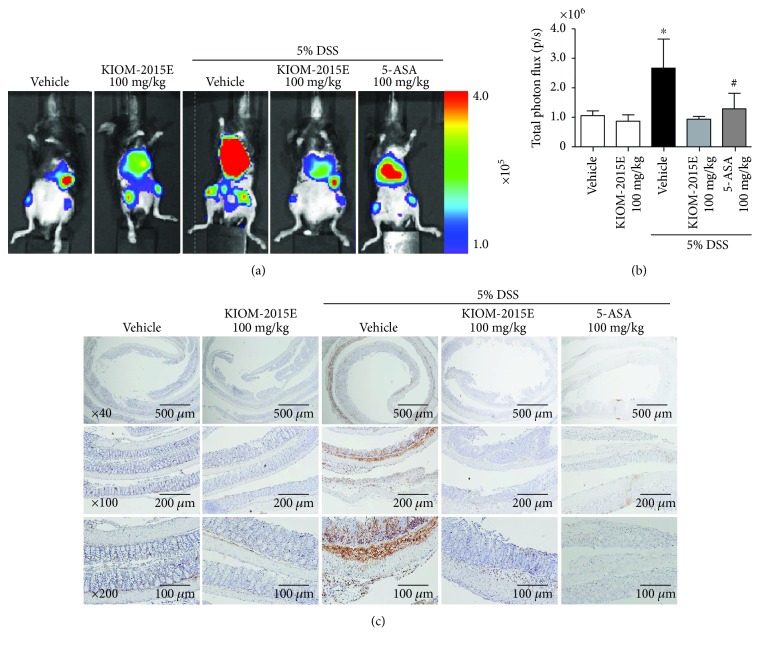
Effects of KIOM-2015E on macrophage infiltration in the dextran sodium sulfate- (DSS-) induced colitis model. (a) Representative image for the *in vivo* imaging system (IVIS). (b) Quantification of total photon efflux from a reporter Raw264.7/NF macrophage that migrated into an inflammation site. On the 7th day of the experiment, the mice were injected in the tail intravenously with 100 *μ*l of Raw264.7/NF cells (3 × 106 cells/mouse). On the 8th day of the experiments, the mice were injected intraperitoneally with 100 *μ*l of D-luciferin (3 mg/mouse). The acquisition of BLI intensity measured using IVIS. (c) Immunohistochemistry for F4/80 antibody. Collected intestinal tissues were fixed with 4% paraformaldehyde solution, embedded in paraffin block, and sectioned using a microtome. Histological sections were stained with anti-F4/80 antibody. The total photon efflux results represent the mean ± standard error of the mean values of each mouse in the same group. ^∗^*p* < 0.05 versus the control group; ^#^*p* < 0.05 versus the DSS-treated group.

**Figure 7:**
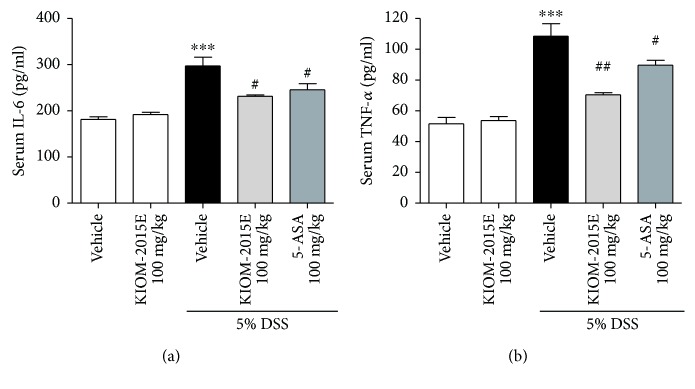
Effects of KIOM-2015E on the serum levels of proinflammatory cytokines in the dextran sodium sulfate- (DSS-) induced colitis model. (a) Serum levels of interleukin-6 (IL-6) (b) Serum levels of tumor necrosis factor-*α* (TNF-*α*). The serum levels of proinflammatory cytokines were detected by ELISA kit for IL-6 and TNF-*α*. Cytokine results represent the mean ± standard error of the mean values of each mouse in the same group. ^∗∗∗^*p* < 0.001 versus the control group; ^#^*p* < 0.05 and ^##^*p* < 0.01 versus the DSS-treated group.

A previous study showed that DSS induced the dysfunction of the intestinal barrier and aggravated the inflammatory process, following the lead to macrophage infiltration into the colon [16]. Therefore, we verified the effects of KIOM-2015E on infiltration of macrophage using RAW264.7/NF cells by the in vivo imaging system. Bioluminescent signals shown as the infiltration of macrophage were significantly increased in the DSS-treated group compared with the control group (Figures 6(a) and 6(b)). However, KIOM-2015E obviously decreased the bioluminescent signal compared with the DSS-treated group (Figures 6(a) and 6(b)). Furthermore, consistent with IVIS results, macrophage as stained by F4/80 antibody was significantly increased in the DSS-treated group compared with the control group (Figure 6(c)). However, KIOM-2015E obviously reduced infiltration of macrophage compared with the DSS-treated group (Figure 6(c)). Several reports showed that proinflammatory cytokines into the serum increased secretion in DSS-induced colitis [17, 18]. Thus, we measured serum cytokine levels of IL-6 and TNF-*α*. Both levels of IL-6 and TNF-*α* were significantly higher in the DSS-treated group than in the control group (Figures 7(a) and 7(b)). However, both levels of IL-6 and TNF-*α* in mice treated with KIOM-2015E were lower than those in the DSS-treated group (Figures 7(a) and 7(b)).

## 4. Discussion

In the present study, we demonstrated that KIOM-2015E inhibited the downregulation of tight junction proteins such as ZO-1 and occludin and improved the dysfunction of the intestinal barrier induced by IL-6 and DSS. Furthermore, we showed that the loss of body weight, intestinal shortening, and crypt damage were reduced by treatment with KIOM-2015E. Increasing productions of proinflammatory cytokine are associated with DSS-induced colitis [17, 18], which produce various proinflammatory cytokines, including TNF-*α*, IFN-*γ*, IL-6, IL-8, IL-12, and IL-17 [17, 19, 20]. Many studies showed the regulation of cytokines as potential targets in therapeutic approaches [21, 22]. Previous reports showed that major compounds of KIOM-2015E identified orientin, isoorientin, and vitexin [23]. Especially, vitexin has anti-inflammatory [24, 25], anticancer [26, 27], and antioxidant properties [28]. Our study showed that KIOM-2015E also reduced the levels of proinflammatory factors TNF-*α* and IL-6 in serum. Moreover, KIOM-2015E inhibited oxidative stress by decreasing MPO activity in colon tissue. Collectively, these results indicated that KIOM-2015E alleviates DSS-induced colitis though regulation of intestinal permeability and inflammation considering the anti-inflammatory and antioxidant effects of vitexin.

Dysfunction of the intestinal epithelial barrier has been one of the major symptoms of IBD, including UC and CD. Also, defective intestinal barrier function has been known as a critical pathogenic factor of inflammatory conditions in IBD [29]. These dysfunctions increased intestinal permeability in a patient of IBD. In this study, we confirmed the expression of ZO-1 and occludin as the representative proteins of tight junctions. It was found that KIOM-2015E repaired the expression of tight junctions and its associated protein ZO-1 and occludin in the treatment and prevention of colitis diseases.

Several reports showed that lymphocytes, macrophages, and neutrophils markedly increased in number in DSS-induced acute colitis [17, 20]. Especially, mainly macrophages could contribute to abnormal production of inflammatory cytokines and mediators [30]. Also, macrophages have an important role in immune responses, in which recruited macrophages are known to be associated with the secretion of TNF-*α*, IL-6, and IL-1*β* [31]. The present study confirmed in the IVIS image that the infiltration of macrophage with bioluminescent signals was increasing in DSS-treated mice, whereas KIOM-2015E significantly decreased the infiltration of macrophage. Furthermore, immunohistochemistry data showed that the macrophages with the F4/80 marker were increased in DSS-treated mice and KIOM-2015E inhibited macrophage infiltration into the colonic mucosa in DSS-induced mice in consistency with IVIS data. Taken together, our results verified that KIOM-2015E relieved inflammation by inhibiting macrophage infiltration into the intestinal crypts in DSS-induced colitis mice.

## 5. Conclusion

This present study proved that KIOM-2015E exerted useful effects by improving intestinal barrier function and inhibiting inflammation in DSS-induced colitis mice. It was also indicated that due to the beneficial effects of KIOM-2015E, its inhibition of macrophage infiltration into the intestinal epithelium is likely. In conclusion, KIOM-2015E is a potentially new therapeutic agent for colitis through anti-inflammatory activity and maintenance of the colonic epithelial barrier.

## Data Availability

All the data supporting the results are shown in the article and can be requested from the corresponding author.
